# Giant crystals inside mitochondria of equine chondrocytes

**DOI:** 10.1007/s00418-016-1516-6

**Published:** 2016-12-24

**Authors:** S. Nürnberger, C. Rentenberger, K. Thiel, B. Schädl, I. Grunwald, I. Ponomarev, St. Marlovits, Ch. Meyer, D. Barnewitz

**Affiliations:** 1 Department of Trauma Surgery, Medical University of Vienna, Waehringer Guertel 18-20, 1090 Vienna, Austria; 2 Ludwig Boltzmann Institute for Experimental and Clinical Traumatology, AUVA Research Center, Donaueschingenstrasse 13, 1200 Vienna, Austria; 3 University Clinic of Dentistry, Medical University of Vienna, Sensengasse 2a, 1090 Vienna, Austria; 4 Austrian Cluster for Tissue Regeneration, Vienna, Austria; 5 Physics of Nanostructured Materials, Faculty of Physics, University of Vienna, Boltzmanngasse 5, 1090 Vienna, Austria; 6 Department of Adhesive Bonding Technology and Surfaces, Fraunhofer Institute for Manufacturing Technology and Advanced Materials, Wiener Strasse 12, 28359 Bremen, Germany; 7 Clinic and Polyclinic for Traumatology, University of Giessen, Rudolf-Buchheim-Straße 7, 35385 Giessen, Germany; 8 Orthopaedic and Trauma Surgery, Klinikum Saarbrücken, Winterberg 1, 66119 Saarbrücken, Germany; 9 Research Centre for Medical Technics and Biotechnology, Geranienweg 7, 99947 Bad Langensalza, Germany

**Keywords:** Cartilage, Protein crystals, Mitochondria, Chondrocytes, Histochemistry, Transmission electron microscopy

## Abstract

The present study reports for the first time the presence of giant crystals in mitochondria of equine chondrocytes. These structures show dark contrast in TEM images as well as a granular substructure of regularly aligned 1–2 nm small units. Different zone axes of the crystalline structure were analysed by means of Fourier transformation of lattice-resolution TEM images proving the crystalline nature of the structure. Elemental analysis reveals a high content of nitrogen referring to protein. The outer shape of the crystals is geometrical with an up to hexagonal profile in cross sections. It is elongated, spanning a length of several micrometres through the whole cell. In some chondrocytes, several crystals were found, sometimes combined in a single mitochondrion. Crystals were preferentially aligned along the long axis of the cells, thus appearing in the same orientation as the chondrocytes in the tissue. Although no similar structures have been found in the cartilage of any other species investigated, they have been found in cartilage repair tissue formed within a mechanically stimulated equine chondrocyte construct. Crystals were mainly located in superficial regions of cartilage, especially in joint regions of well-developed superficial layers, more often in yearlings than in adult horses. These results indicate that intramitochondrial crystals are related to the high mechanical stress in the horse joint and potentially also to the increased metabolic activity of immature individuals.

**Electronic supplementary material:**

The online version of this article (doi:10.1007/s00418-016-1516-6) contains supplementary material, which is available to authorized users.

## Introduction

Protein crystals are dense aggregations of macromolecular protein units which either form a periodic inner substructure or have a geometric shape, or both. In electron optic images, protein crystals appear evenly electron dense, with either a grid or a dot structure or regularly arranged lines, ladders or helices, and sometimes they have sharp straight borders. At cellular level, they are well known from the granule in mature eosinophil immune cells (Acharya and Ackerman 2014), and they have also been described in several further cell types from prokaryotes to eukaryotes, including bacteria (Bechtel and Bulla 1976), yeast (Veenhuis et al. 2003), invertebrates (Pabst et al. 2006), vertebrates (Djaldetti and Feller 1978; Nagano and Otsuki 1971; Ward 1962) and plants (Moura et al. 2010).

Protein crystals function as storage structures in highly metabolic active tissues such as specialized secretory cells (Santos 1966), hormone-dependent cells (Hawkes 1993; Mesa et al. 2015) or toxin-producing cells (Bechtel and Bulla 1976). They also function under conditions of reduced metabolism and starvation (Ericsson et al. 1966; Hamilton et al. 1966). Further, they are found under pathologic conditions, for instance in cancer (Dogan et al. 2012) or ischaemia (Hanzlikova and Schiaffino 1977). Crystals have been described in almost all parts of the cell: in metabolic active cell compartments as the endoplasmic reticulum (Hasegawa et al. 2011; Jones et al. 1999; Koopmann et al. 2012), secretory vesicles (Santos 1966) and the cytoplasm (Kozina et al. 2011; Schonherr et al. 2015) but also at sites of degradation, the lysosomes (Tsutsui et al. 2015). Additionally, crystals may appear in mitochondria (Farrants et al. 1988), peroxisomes (Schonherr et al. 2015) and the nucleus (Gouranton and Thomas 1974). Typically, a specific crystal type is found in a specific subcellular compartment and is not ubiquitously distributed within the cell, although there are some exceptions (Jain et al. 2001). The protein components may, however, derive from other cell compartments and be transported to the site of crystallization, as it has been observed in the midgut of beetles, where proteins were synthesized in the cytoplasm and transported into the nucleus, where crystals are formed (Gouranton and Thomas 1974).

Little is known about the composition and function of crystals formed in several environments, since the exact identification of the proteins requires the isolation of a certain amount of material, which appears to be difficult in terms of techniques, quantity and frequency. Many crystals are very small, rare or unpredictable in their frequency; some even appear as single observations (Klepal et al. 2010; Silvestro and Chapman 2004). There are, however, examples of adequate descriptions of crystals in bacteria, in cases with less challenging methodological conditions (Sawaya et al. 2014) or in which crystals could be artificially induced, for example via genetic manipulation (Sawaya et al. 2014; Schonherr et al. 2015; Thamwiriyasati et al. 2010; Tsutsui et al. 2015) or by means of free-electron laser and serial femtosecond crystallography (Sawaya et al. 2014).

The best characterized crystals in vertebrates are the granule in mature eosinophil immune cells (Acharya and Ackerman 2014) and Reinke’s crystals in testes, which were first described in 1896, mainly in Leydig cells (Reinke 1896). Several studies have confirmed that Reinke’s crystals are composed of steroidogenic enzymes and appear only in mature testes and testosterone-producing men, mainly in cryptorchid patients (Kozina et al. 2011). The suggested differences between tumour and non-tumour could, however, not be confirmed. The lack of Reinke’s crystals in some studies has been methodologically induced, since they degrade during water-based formalin fixation and require fixation in absolute alcohol to be preserved (Mesa et al. 2015).

In the present study, crystals were found in formalin- and glutaraldehyde-fixed samples in giant mitochondria of chondrocytes. They were extensively analysed with different morphological methods such as transmission electron microscopy, element and Fourier transform analysis and histochemical staining. Even though the chemical composition is not yet clarified, the identification of crystal localization has led to hypothesize a relationship between crystal development and mechanical stress conditions.

## Materials and methods

### Sample generation

The main part of this study was performed on native articular cartilage control samples from a horse study and the native cartilage surrounding of an experimentally treated cartilage area. The study was on cartilage defect treatment of experimental full thickness defects of 1.5 × 2.0 cm set in the trochlear ridge of the distal femur without damaging the subchondral bone. Defects were treated with four different biodegradable biomaterials seeded with autologous cells gained eight weeks before from the talocrural joint (Nürnberger et al. 2013a) and further with a scaffold-free transplant produced in vitro under mechanical stimulation (Ponomarev and Wilke 2004). Five yearlings were treated in a pilot study with cell-loaded Hyalograft^®^ (Fidia, Italy), Bio-Gide^®^ (Geistlich, Germany) biomaterials and the scaffold-free construct. Study time was three months. Nineteen adult mare horses were transplanted with cell-loaded Hyalograft^®^, Geistlich^®^ and CaRes^®^ (Arthro-Kinetics, Austria) biomaterials and the scaffold-free construct. The endpoint of the study was one year after implantation. After surgery, the horses stayed two weeks in boxes, were then exercised (walking) and kept thereafter at paddocks. The horses used for this study were healthy Haflingers that did not meet breeding criteria. The study was verified and approved by the local ethical board (Thüringer Landesamt für Lebensmittelsicherheit und Verbraucherschutz Nr. 14-03/03, Germany).

Further samples of equine articular cartilage of one adult and two juvenile horse (1 year and 3 months) from different breeders and races and hoof cartilage of 6 adult and one juvenile horse, harvested according to the rules of good scientific practice and approved by the ethical committee, were kindly provided from the University of Veterinary Medicine, Vienna. Sections of articular and epiphyseal cartilage of other species (articular cartilage of rat, chicken, sheep, calf; metaphyseal cartilage of rat and pig) were kindly provided from collaborating laboratories (Ludwig Boltzmann Institute for Experimental and Clinical Traumatology, Vienna, Austria). Human cartilages from femoral heads after femoral head fracture, taken from intact non-arthritic areas, were available in our laboratory from previous investigation (Nürnberger et al. 2006b). Juvenile human cartilage harvested after surgical extraction of supernumerary fingers of polydactyly patients was provided from the Department of Orthopaedics, Medical University of Vienna. Informed consent was obtained from all individual participants included in the study.

### Sample preparation and fixation

Articular cartilage from the horse was fixed either for histology or for electron microscopy. Histological samples were immersed in 7.5% buffered formaldehyde, rinsed, dehydrated and embedded in paraffin. Sections of 4 µm were performed with a microtome (HM355S Micros, Austria). For ultrastructure, samples were fixed in 2.5% glutaraldehyde in 0.05 M sodium cacodylate (pH 7.3). Samples were then postfixed with 0.5% osmium tetroxide containing 1% potassium ferrocyanide and dehydrated in a graded series of alcohol and embedded in Agar 100 or low viscosity resin. Semithin sections (1 μm) were cut with an ultramicrotome (UC7 Leica Microsystems, Austria), stained with toluidine blue and imaged with an E800 Nikon laboratory microscope.

### Transmission electron microscopy (TEM)

Ultrathin sections (70 nm) of resin blocks were contrasted with uranyl acetate and lead citrate and imaged with a Zeiss 902 (Oberkochen, Germany) or FEI Morgagni (Hillsboro, USA) electron microscope. To further characterize the crystal composition, TEM measurements were carried out using a FEI Tecnai F20 S-TWIN microscope (Hillsboro, USA) equipped with a GATAN imaging filter (GIF2001). The microscope was operated at an accelerating voltage of 200 kV with a field-emission gun (FEG) resulting in a point resolution of 2.4 Å.

The TEM and energy-filtered images were recorded with the slow-scan CCD camera integrated in the GIF (1024 × 1024 pixel array). No binning was used for the TEM images, while for the energy-filtered images a binning of 2 × 2 was applied, so that images with a size of 512 × 512 pixels were generated. Element maps were recorded using the three-window method; drift between successive images was corrected by a cross-correlation algorithm (Hofer et al. 1997).

For the analysis of crystal morphology and structure a Philips CM200 transmission electron microscope (Eindhoven, Netherlands) was used at an accelerating voltage of 200 kV equipped with a Gatan™ Orius CCD camera. Different zone axes of the crystalline structure were obtained by tilting using a Philips double tilt sample holder. The analysis of the crystalline lattice and their structural parameters was carried out by fast Fourier transformation (FFT) of the lattice-resolution images.

### Histochemistry

Different histochemical methods were applied in order to find out whether crystals stain in paraffin sections. First, the unspecific toluidine blue staining was used as for resin sections (0.1% in 2.5% Borax pH 9) (Pease 1964). Then, protocols were tested with various dyes and affinity to acid structures such as nuclei (Movat, Masson, AZAN, HE), basic proteins (Biebrich Scarlet), carboxylated glycosaminoglycans (Alcian blue, Safranin O) or polysaccharides (PAS). Further, special stainings for specific structures such as amyloids (Congo red), bacteria (GRAM), granulocyte granule (Giemsa) and birefringent structures (Picro Sirius Red) were performed.

Movat is a pentachrom staining (12016 Morphisto/Sanova), and Masson Goldner (100485 Merck) a trichrome staining, both with acid fuchsin as cell and nuclei stain. The latter was adapted according to Kozina et al. (2011) by extended acid fuchsin and reduced light green incubation time. Heidenhain’s AZAN was used with the nucleus stain azo carmine (Kiernan 1999) and Martius-Scarlet-Blue (MSB) with Brilliant crystal scarlet apart from Martius Yellow and Methylene blue (Bancroft and Gamble 2008). Hematoxylin and eosin stain (HE) was performed according to the laboratory standard. Periodic acid–Schiff (PAS) (Romeis 1989) is based on basic fuchsin staining (X900 Roth) after oxidative reaction (HP00 Roth). Alcian blue was used as glycosaminoglycan stains to stain carboxylated glycosaminoglycans at pH 2.5 (Romeis 1989) and Safranin O as general glycosaminoglycans stain based on saffron with fast green counterstain. Biebrich Scarlet (0.04%) was prepared according to the original protocol (Spicer and Lillie 1961) using Laskey’s glycine buffer at pH 8.0 and pH 10.0, Gram staining with Carbol-Gentianaviolet solution and Carbol Fuchsin (HP02.1 Roth) and Giemsa (Sigma Aldrich, GS500). Congo red (101340 Merck) and Picro Sirius Red (Whittaker 1995) were analysed under the polarization microscope giving a colour shift to green or green to orange and red, respectively.

Overview sections were scanned with an Olympus BX50 and dotSlide 2.5 software (Olympus) and higher magnifications were imaged with an E800 Nikon research microscope and a NIS Elements software (BR 4.20.00). Manual Z-stacks were performed and combined in Photoshop CS5 (Adobe Systems).

## Results

**Fig. 1:**
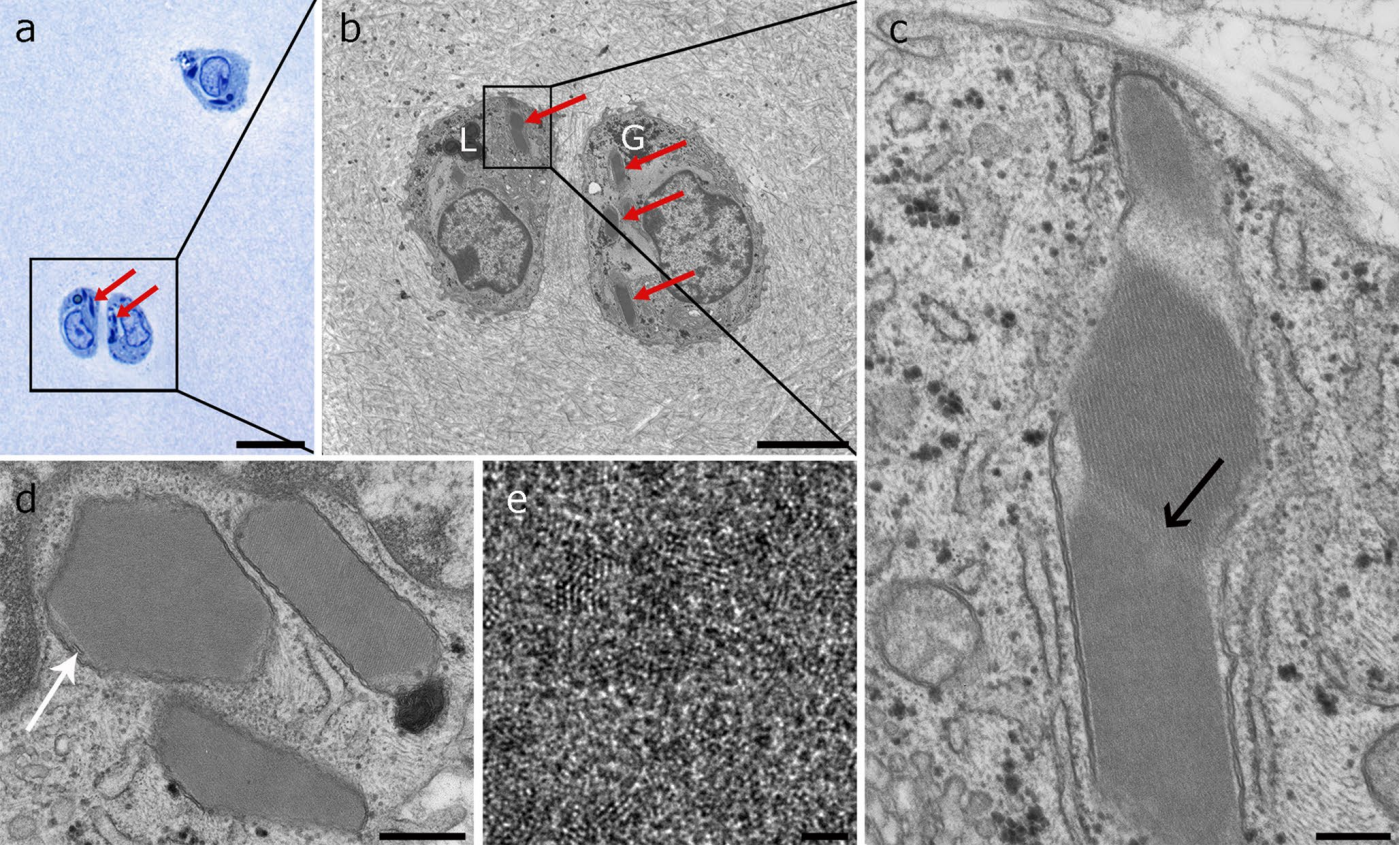
Ultrastructure of intramitochondrial crystals in articular chondrocytes of juvenile horses. **a** Semithin resin section stained with toluidine blue showing three chondrocytes containing two types of intensively stained structures inside the cytoplasm: spherical lipid droplets and crystals (*arrows*). The *square* indicates the area of **b**. **b** TEM image showing the same double chondron as in **a** bearing electron dense lipid droplets (L) and glycogen (G) and protein crystals (*arrows*). The latter have elongated and homogenous structures and a slightly darker appearance than the cytoplasm. The cell on the right contains several crystal transections. The *square* indicates the area of magnification of image **c**. **c** Crystal consisting of three compartments. A striated substructure is visible in the middle compartment. A less dense striated zone forms the transition to the lower compartment (*arrow*). The whole crystal is surrounded by a double membrane. **d** Three crystals with different geometric shapes surrounded by a double membrane. In the outer left-hand crystal, cristae are visible between the crystal and the mitochondrial membrane (*arrow*). **e** High magnification of the crystal structure showing the individual protein units. *Scale bars* are **a** 20 µm, **b** 4 µm, **c** 250 nm, **d** 500 nm and **e** 2 nm

**Fig. 2:**
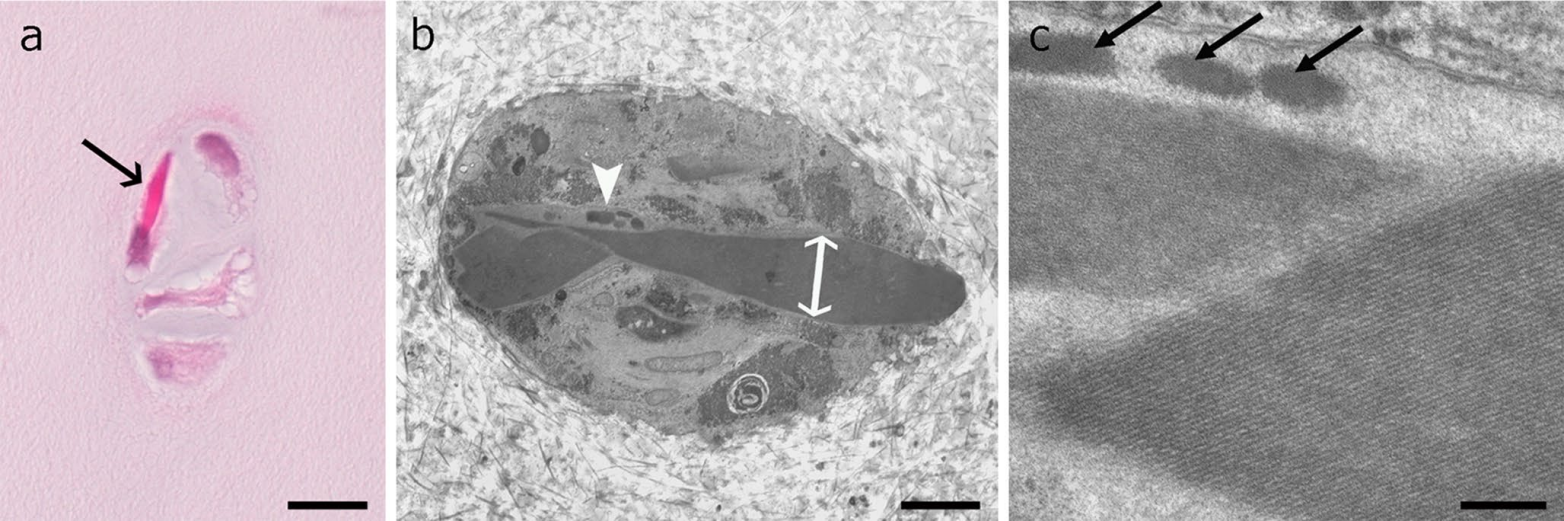
Intramitochondrial crystals in adult horse chondrocytes adjacent to a defect area. **a** Hematoxylin–eosin-stained chondron of four chondrocytes form a small-sized cluster. One cell contains an intensively stained crystal of about 3.5 µm in diameter (*arrow*). **b** TEM image showing several crystal transections in one cell with a maximal diameter of 2 µm (*double arrow*) and extending the cell membrane by stretching beyond the natural limit of the cell. Adjacent to the large crystal homogenous dense spots indicate nucleation centres of new crystals (*arrowhead*). **c** Higher magnification of the interface between two crystals and three nucleation centres of further crystals (*arrows*). Note that the crystal on the left-hand outer side has a slightly striated substructure. *Scale bars* are **a** 20 µm, **b** 2 µm and **c** 200 nm

**Fig. 3:**
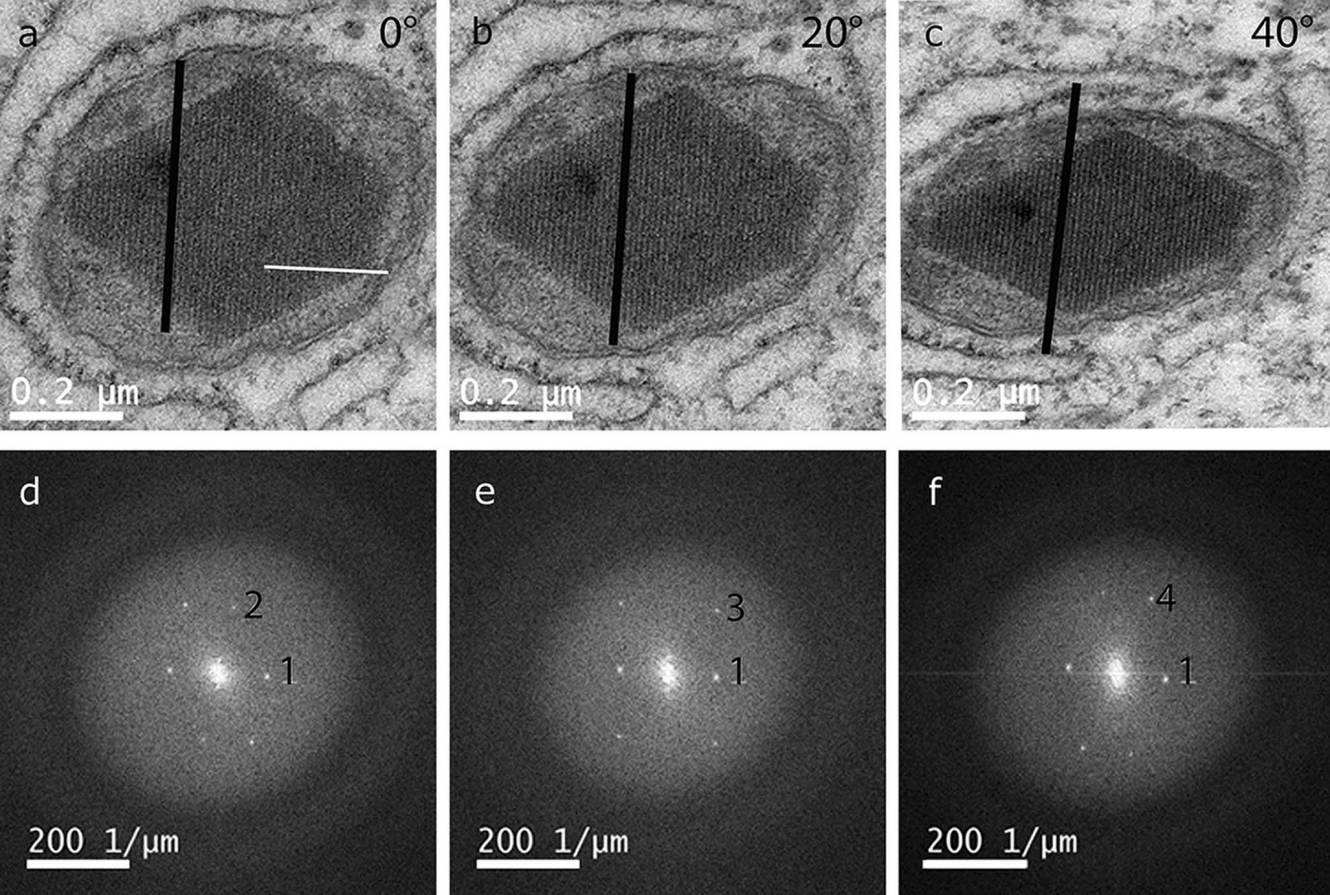
Tilted TEM images and Fourier transform. **a**–**c** TEM lattice plane images of a crystal in mitochondria taken at different orientations (**a** 0°, **b** 20°, **c** 40°) by systematic tilting around the *white line*. While one plane (parallel to the *black line*) is kept edge on, the 2-dimensional projections show different periodic structures. **d**–**f** The corresponding Fourier transform images of the electron micrographs show several spots whose distance from the centre is inversely related to the lattice plane spacing. Due to the systematic tilting, spot 1 is present in all three images

**Fig. 4:**
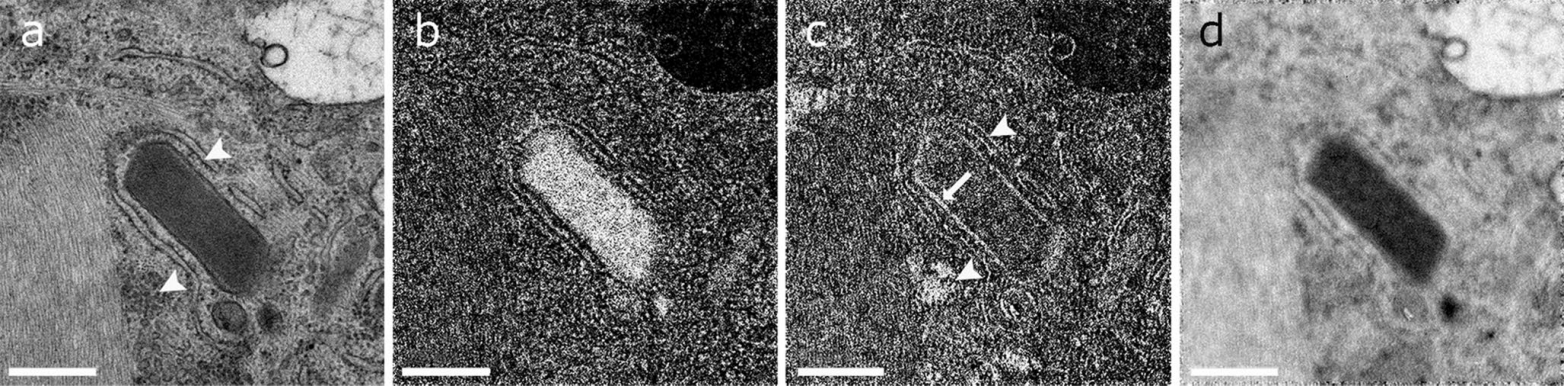
Elemental distribution analysis of the crystals with EFTEM. **a** The zero-loss image displays the overall structure of the crystal. *Arrowheads* indicate ribosomes at the ER and in the cytoplasm as also indicated in **c**. **b** Nitrogen is clearly detected in the crystalline structure. **c** Sulphur is not positive in the crystal but the surrounding mitochondrial plasm (*arrow*) and the ribosomes at the ER and in the cytoplasm (*arrowhead*). **d** Phosphor did not show any signal inside the crystal. *Scale bars* are 0.5 µm

**Fig. 5:**
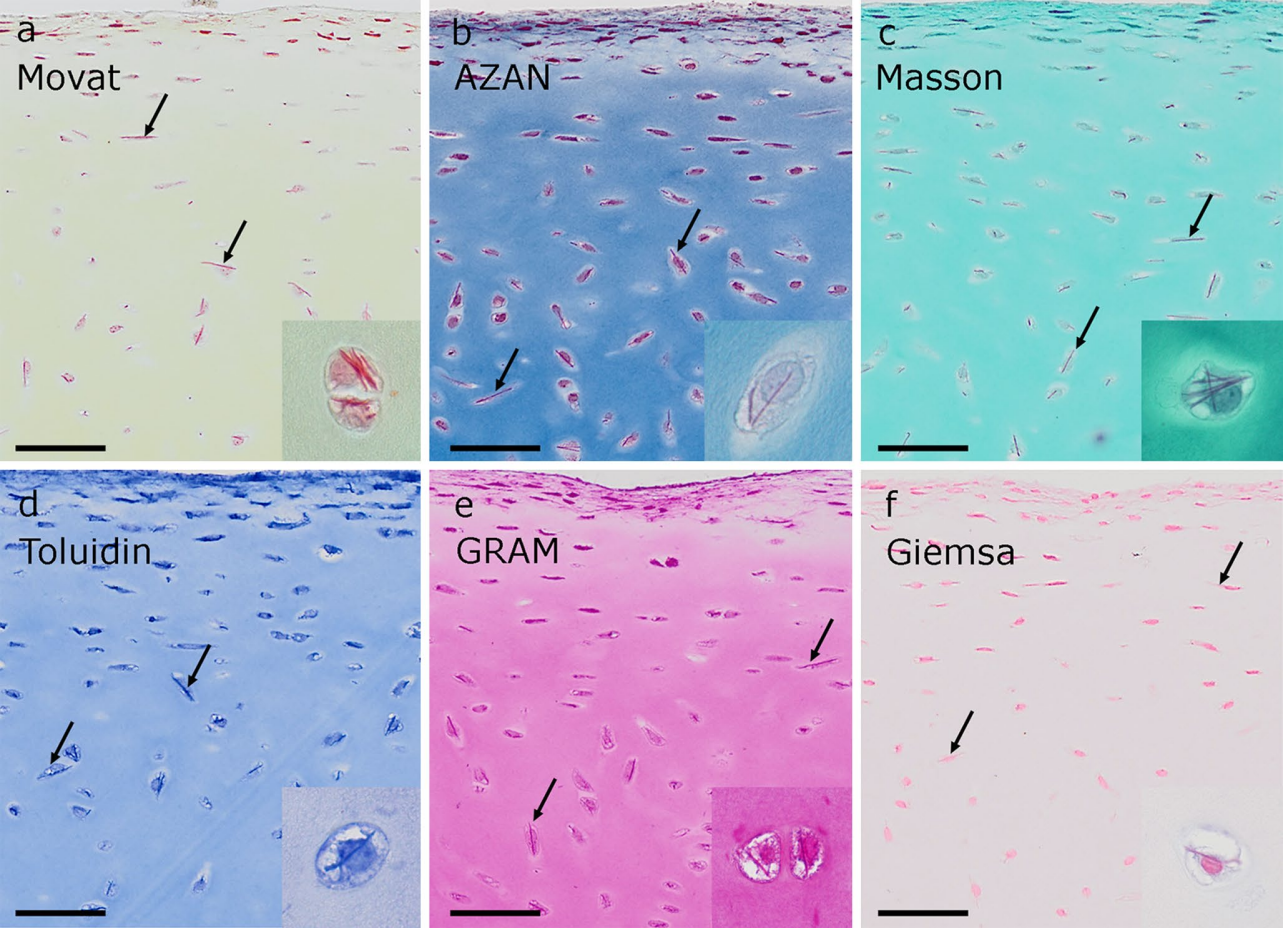
Histochemical methods that intensely stain the crystals in paraffin sections. The *arrows* indicate two of the most striking crystals in each overview image appearing as intensively stained needles in faintly stained cells. **a** Movat, **b** AZAN and **c** Masson stain the cellular matrix less intensive than the crystals, resulting in a clear visualization of the crystals even in overview images. It is obvious that the crystals stretch throughout the whole cells and are orientated along their long axis and therefore in the superficial zone parallel to the joint surface. The detail images (*insert*) show several crystals in a single cell. **d**–**e** In toluidine blue and GRAM, the intensity of the matrix impedes a clear discrimination of the crystals in overview images, even though the detail images (*insert*) show that the crystals are stained. **f** In Giemsa, the staining of both, cytoplasm and crystal, is quite pale. *Scale bars* are 50 µm

In the course of a horse study on tissue-engineered cartilage regeneration, native cartilage of transplanted as well as that of control animals was analysed. In the course of these investigations, in both treated and control animals, crystals were observed in ultrathin sections in the transmission electron microscope (Figs. 1, 2, S1) and analysed with Fourier transform (Fig. 3) and element analysis (Fig. 4) in order to prove their protein crystalline character. For additional explorations on their distribution in the cartilage tissue and throughout different species, paraffin sections were screened using several stainings to further provide a rough chemical classification (Figs. 5; S2).

### Transmission electron microscopy

Transmission electron microscopic images revealed the overall size of the crystal and information about its inner structure. They had an elongated outer morphology, stretching throughout the whole cell (Figs. 1, S1a) over several µm length and sometimes deforming the cell membrane when the crystal extended the limit of the cell (Fig. 2b). In cross section, they had a geometrical profile with various shapes. Typical geometric structures were hexagons with four long and two short sides (Figs. 1d, 3, S1b) or elongated hexagons with two long and four short sides (Figs. 4, 9c). Further, pentagonal or undefinable cross section structures were found. With higher magnification, the regular crystal structure became obvious (Figs. 1c, d, 2c, 3, S1b) with parallel lines in a distance of 10 nm. In cross or oblique section and higher magnification tightly aligned 1–2 nm granular units emerged (Fig. 1e).

In general, some crystals seemed to consist of several subunits, placed next to each other (Fig. 1c). Higher magnification revealed that they merge together by connecting crystal planes. Those subunits may have different orientations and therefore varied in the appearance of the crystalline substructure (Figs. 1c, 2c).

TEM images further revealed that the crystals were located inside mitochondria since they were surrounded by a double membrane bearing cristae in the narrow remaining space between crystal and double membrane (Fig. 1d). Occasionally the cristae were arranged to untypical network-like structures (Fig. S1c). The crystals completely filled the intramitochondrial space and seemed to stretch the mitochondrion to the length of the crystal. Non-crystal bearing mitochondria found in the same cells had normal size (200–500 nm in diameter).

A single mitochondrion could also have several separate crystals lying in parallel (Fig. S1b). Apart from typical crystals, deposits of a homogenous substance could be seen inside crystals-containing mitochondria. They were small with some hundred nanometre; they had a roundish shape and indefinite edges (Fig. 2b, c). A marginally darker contrast than typical crystals could be observed, and in some a slight striation was discernible. Overall, they appeared like premature crystals.

The crystalline parameters were further characterized with lattice analysis in TEM and Fourier transformation (Fig. 3). The intramitochondrial crystals were imaged along three different zone axes by tilting around a defined axis (Fig. 3a white line) with a tilt angle of 20° and 40° (Fig. 3b, c) with respect to the plane image (Fig. 3a). The Fourier transform images (Fig. 3d–f) show sharp spots, whose position and inverse distance from the centre are correlated to the orientation and spacing of the corresponding lattice plane, respectively. While distances and angles in the plane of projection can be measured accurately at small tilts of the sample, the uncertainty increases with the increased tilt angle. In addition, since spots in the FFT image are visible over a large tilt range (about 10°) due to the extended intensity of the spots in the direction of projection, the error of angles in the direction of projection is ±3°. Therefore, only a list of characteristic spacing and angle α of lattice planes can be given (Table 1).

**Table 1:** Lattice plane spacing and angle α between planes represented by the spots denoted 1–4 in Fig. 3d–f

Spot #	1	2	3	4
Lattice spacing (nm)	10.4 ± 0.2	7.7 ± 0.2	6.1 ± 0.2	6.5 ± 0.3
Angle α (1;#) (°)	0	80 ± 1	56 ± 2	72 ± 3

Energy-filtered TEM (EFTEM) images were carried out to measure the composition of the crystals and the surrounding material. The protein relevant elements nitrogen (*E*_*K*_ = 401 eV), sulphur (*E*_*L*2,3_ = 165 eV) and phosphor (*E*_*L*2,3_ = 132 eV) were acquired (Fig. 4). Inside the crystals, nitrogen was increased, while sulphur could only be measured on the crystal surrounding membrane and in the cytoplasm. Phosphor was absent in the crystal.

### Histology

 Using light microscopy of toluidine blue-stained semithin resin sections, crystals were stained intensively and identified within the same cells as in TEM (Fig. 1a). Therefore, it was tested whether the crystals were also stained in paraffin sections, which were formalin-fixed in contrast to the glutaraldehyde and osmium tetroxide treatment used for resin sections. Using toluidine blue staining, it was equally possible to visualize the crystals in paraffin sections (Fig. 5d). In order to get more information about the histochemical properties, fourteen stainings were applied to native equine cartilage. The dyes were targeted against proteins, sugar and mucus substances (Figs. 5, S2). Crystals retained the dyes of general histological stains and appeared red, magenta or purple with Movat, AZAN, Masson, GRAM, and Giemsa and the unspecific toluidine blue (pH 9.0) staining (Fig. 5). Moderate staining was found using MSB and HE (Fig. S2a, b). Faint or no staining was evident with PAS (for sugars), Alcian blue, Safranin O (glycosaminoglycans) and Biebrich scarlet at pH 8.0 and 10.0 (basic proteins) (Fig. S2c–f). In the polarization filter microscope, the crystals did not show any birefringence or colour effect with Picro Sirius red-stained cells or any green colour effect with Kongo red (data not shown). Out of the tested dyes, Movat and AZAN (Fig. 5a, b) gave the brightest staining and best contrast to the surrounding tissue staining (Table 2).

**Table 2:**
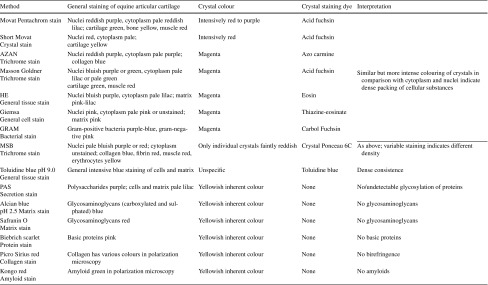
Stainings used for crystal detection and rough identification of its nature

**Fig. 6:**
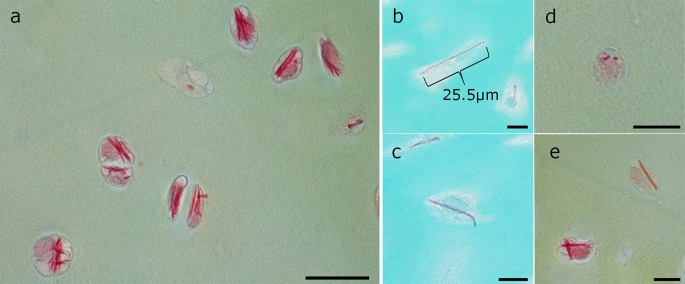
Several examples of different numbers and appearance of crystals inside chondrocytes of native articular cartilage. Details of Movat (**a**, **d**, **e**) and Masson Trichrom (**b**, **c**) stained cartilage areas with crystals in almost all cells. **a** Cells contain several crystals arranged in bundles and aligned the long axis of the cell in elongated chondrocytes. **b** Crystal stretching through the whole cell measuring 25.5 µm. **c** Chondrocyte containing acrystal with bent end. **d** In cross section through the crystals, they appear as intensive red dots. **e** Chondrocyte with crossed crystal bundles and a faintly stained cell with a slightly orange crystal. *Scale bars* **a** 20 µm, **b**–**e** 10 µm

**Fig. 7:**
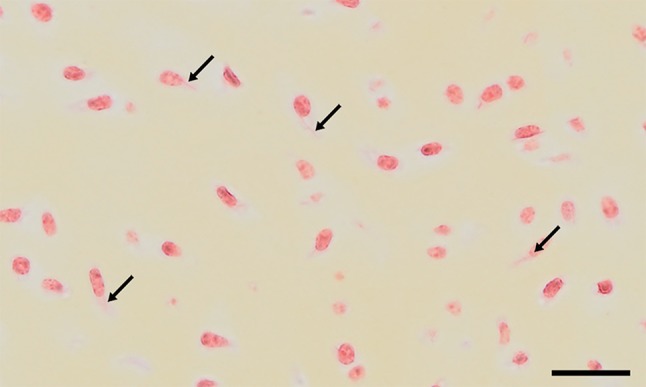
Cartilage of a 3-month-old foal stained with “crystal-stain”, a short version of Movat based on acid fuchsin and saffron. Several cells bear crystals (*arrows*) stretching throughout the whole cell. *Scale bar* 50 µm

**Fig. 8:**
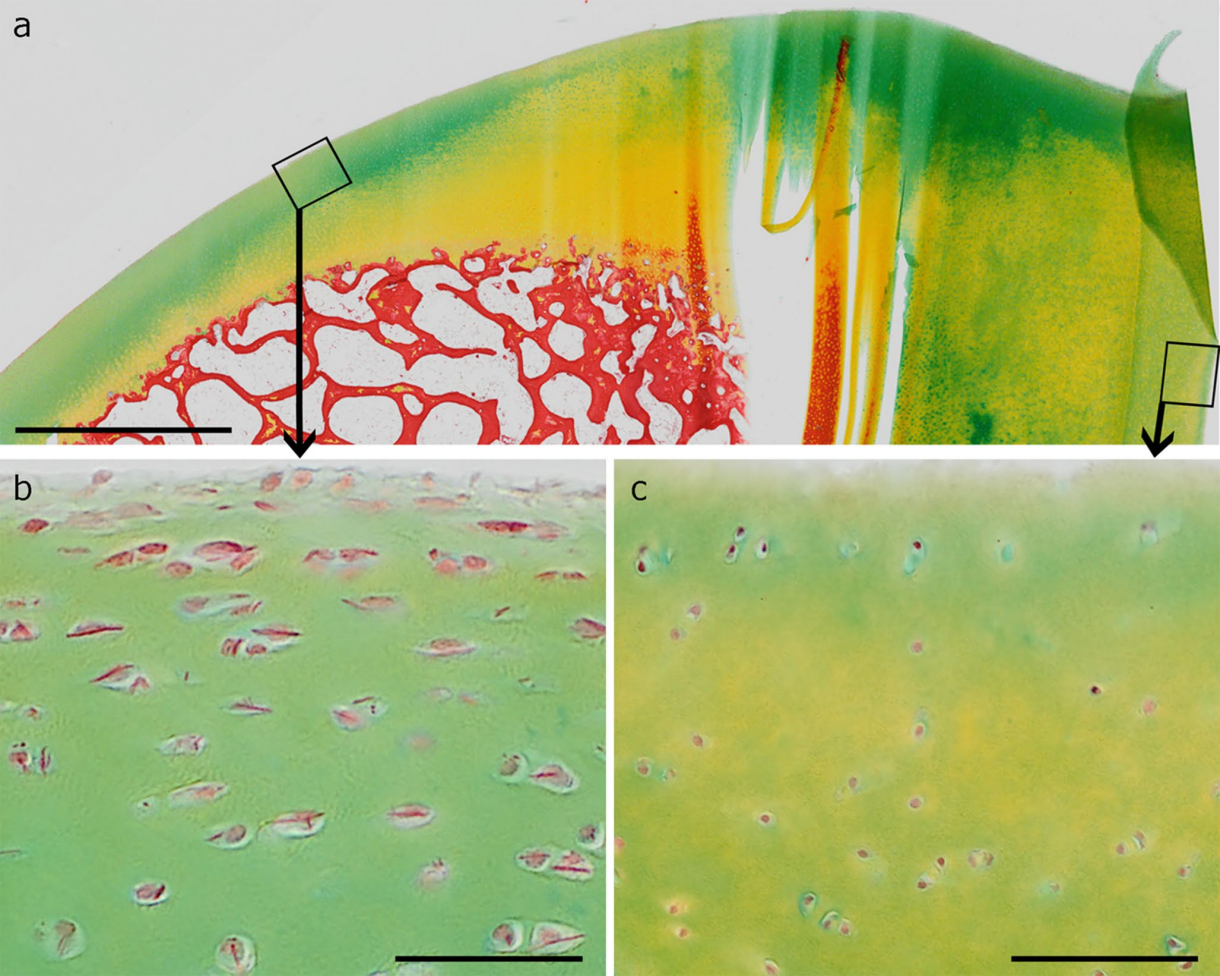
Movat staining of native articular cartilage with several morphological regions correlating with different frequency of crystals. The *squares* in the overview (**a)** indicate the area of magnification in **b** and **c**. **b** The thinner cartilage region with elongated chondrocytes in the superficial layer contain chondrocytes with crystals in almost every cell, whereas no crystals are visible in the thick cartilage region with mainly spherical chondrocytes up to the cartilage surface (**c**). *Scale bars* **a** 2 µm, **b** and **c** 50 µm

An analysis of the crystals in paraffin sections confirmed the electron microscopic observation that they were needle-like and stretching through the whole cells (Figs. 2a, 5, 6, 7, 8b, S3c) measuring up to 25 µm (25.5 µm was the largest measured; Fig. 6b). Some crystals appeared tilted or branched (Fig. 6c), probably due to the fusing crystals seen in TEM. As in TEM, several crystals could be distinguished within one cell; they were more or less orientated along the axis of the cells (Figs. 5, 6, 7, S3). Therefore, the orientation of the elongated crystal also showed a specific pattern in the tissue corresponding to the orientation of the cells. If transversely cut, the crystals were discernible as dense homogenous spots (Fig. 6d). In pale degrading cells, also the crystals change their colour from intensive red to orange, indicating that the crystal proteins altered during decay (Fig. 6e).

In order to screen the presence and distribution of crystals, a short version (“crystal-stain”) of the most intensive Movat-stain was created based on Acid Fuchsin for the crystals and saffron for a faint matrix colour. This stain was then used for screenings of other species: articular cartilage from chicken, rat, pig, sheep and human and epiphyseal cartilage from rat and pig. None of those species bore crystals inside chondrocytes. However, amongst articular cartilage samples of one adult and two juvenile horses of another source, crystals were found in a 3-month-old foal (Fig. 7).

The distribution of crystal-containing chondrocytes inside the articular cartilage tissue was characterized in samples taken from various regions of the equine knee joint. Crystals were either all over the three non-calcified cartilage zones (superficial, middle and deep zones) or preferentially in the superficial and transition zones (Figs. 5, 8 and S3). They were rare in the deep zone and never detected in the calcified zone, the bone and the periost (Fig. S3). In some regions, there were only single cells containing a single crystal within the whole cartilage transection. Cartilage regions with a well-developed superficial zone with elongate chondrocytes orientated parallel to the joint surface, contained more crystals than areas with spherical chondrocyte appearance even close to the cartilage surface (Fig. 8). This was also found within one and the same cartilage samples (Fig. 8). With frequency of crystal-containing chondrocytes, also the amount of crystals per cell increased (Fig. 6). The highest amount of crystals identified in a cell was six.

**Fig. 9:**
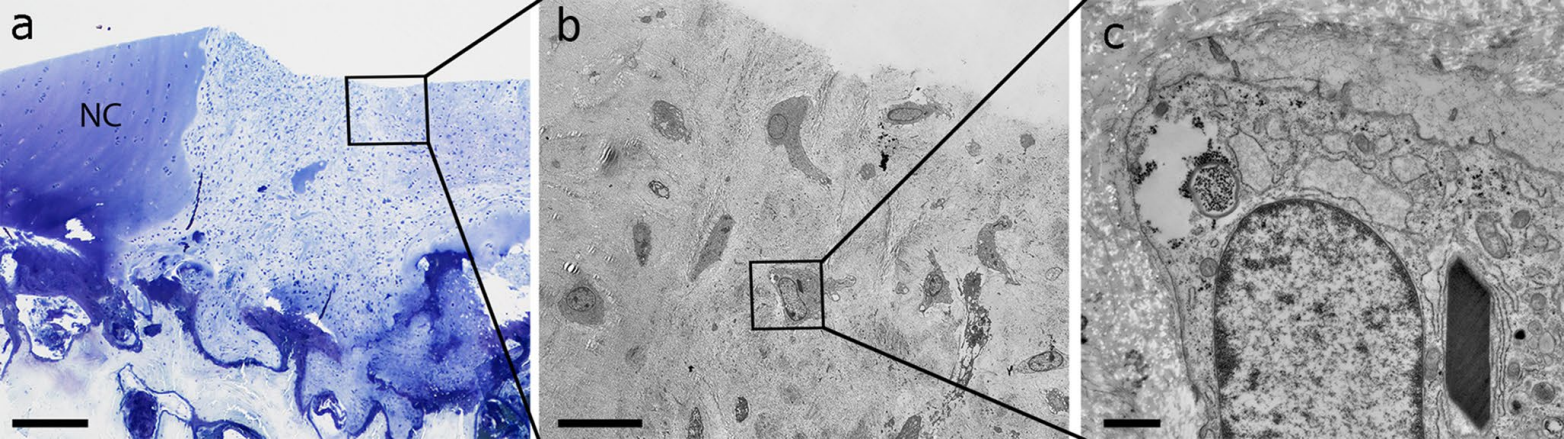
Defect area and regenerative tissue of an experimental cartilage defect in a yearling horse. It was treated with a scaffold-free chondrocyte construct produced under mechanically stimulation in vitro. **a** Toluidine blue-stained overview image showing the transition zone of native cartilage (NC) and regenerative tissue in the defect area. The *square* indicates the area shown in the TEM image (**b**). **b** The TEM image shows polygonal and spherical cells inside the tissue surrounded by dense matrix. The* square* in **b** indicates the cell which is enlarged in **c**. **c** A rhomboidal crystals is visible inside the cell. *Scale bars* **a** 250 µm, **b** 20 µm and **c** 1 µm

In the samples of the Haflinger horses from the cartilage regeneration study, all five yearlings bore crystals, while in the adult individuals crystals were less frequent and were found in three out of 19 horses. In two of the latter animals, larger and more intensively stained crystals (Fig. 2) were found in restricted areas adjacent to the defects. The mitochondria with the crystals also contained several dark homogenous nucleation centres indicating the formation of further crystals (Fig. 2c). Those areas were in both samples adjacent to the defect area. We also screened the regenerative tissue found in the defect areas: In the group treated with scaffold transplants, no crystals were present. In the transplantation site, crystals were present only in the regenerative tissue, which developed from scaffold-free transplant based on equine chondrocytes grown under mechanical stimulation (Fig. 9).

## Discussion

In the current study, giant crystals inside mitochondria of equine articular cartilage chondrocytes were analysed with morphological methods and results were compared with data of the current literature. Most literature data of intramitochondrial crystals in general are from the booming period of ultrastructure, the 1970s, which is probably due to the fact that crystal formation in mitochondria could not be experimentally manipulated, as it is the case for organelles such as ER and peroxisomes (Sawaya et al. 2014; Schonherr et al. 2015; Tsutsui et al. 2015). Therefore, the main information available is on ultrastructure.

At any rate, there are many descriptions of intramitochondrial crystals, especially in lower organisms such as protozoa (Suganuma and Yamamoto 1980) and invertebrate (Davis 1967; Hawkins et al. 1980) but also vertebrate (Hawkes 1993; Ward 1962) including Mammalia (Saito and Fleischer 1971; Shiraki and Neustein 1971). Those crystals have either only an inner crystalline structure (Bhagwat and Ross 1971; Saito and Fleischer 1971) or also a crystalline outer shape (Suganuma and Yamamoto 1980; Ward 1962). They appear in different cell types but with higher frequency in liver and muscle under pathological conditions such as ischaemia (Hanzlikova and Schiaffino 1977; Shiraki and Neustein 1971; Swenson et al. 1967), protein deficiency or starvation (Ericsson et al. 1966; O’Gorman et al. 1997; Taira 1979) or intoxication with alcohol (Voelz 1968) and different types of myopathies (Farrants et al. 1988).

The current study is to our knowledge the first one characterizing extensive protein crystals in mitochondria of chondrocytes. In one publication describing the ultrastructure of equine cartilage, crystals were mentioned and shown in ultrastructural images of equine chondrocyte (Horký and Tichý 2002) but without any description of their subcellular localization and structure. Shape and size of those crystals were the same as in this study, and samples were also taken from articular cartilage of horse. Crystals in chondrocytes have never been described in other species, not even in the studies on canine and feline cartilage performed by the authors who described the presence of crystals in horse chondrocytes (Horký and Tichý 1995, 2004). In order to verify the absence of crystals in other than equine articular cartilage, this study encompassed a screening of cartilage from various species, including different cartilage types (articular, epiphyseal) and ages (skeletal immature vs. adult) based on histological sections. No crystalline inclusions were identified neither in chicken, rat, pig, sheep, calf nor in human. However, when screening horse cartilage harvested and processed in other institutes, foal cartilage also showed mitochondrial crystals. This matched well with the observations made in the samples of the main study that crystals appeared with higher frequency in juvenile horses than in adults.

As in the present study, intramitochondrial crystals in other cell types have been described to be frequently found in giant mitochondria (Bhagwat and Ross 1971; Ghadially and Parry 1966). In contrast to the crystals in the equine chondrocytes, those crystals were clearly smaller than the mitochondria, frequently compartmented and occupied only a part of the organelle. They did not seem to be the reason for the size increase of the mitochondria. On the contrary, in the equine chondrocytes the increasing size of the crystals may be the obvious reason for the enlargement of the mitochondria, since the crystals completely fill the intramitochondrial space and stretch the mitochondrion to an enormous size, sometimes to the full length of the cell. Further, in horse chondrocytes, giant mitochondria only appear in relation with giant crystals. Some crystals were perfectly straight and continuous, suggesting that they developed by continuous growth; others were angulated and surrounded by a mitochondrial membrane that appeared to be segmented, indicating that the size increase occurred by fusion of several mitochondria. Fusion of mitochondria is a normal physiological process and renewal strategy for exchange of information and material (Pernas and Scorrano 2016; Westrate et al. 2014). It is conceivable that in chondrocytes, the growing mitochondria merge at a certain point due to steric reasons and fuse.

Previous studies have suggested that crystals may be caused by retard fixation (Pena 1980) due to disintegrating proteins and lipids during autolysis. Crystals were not found when tissues were fixed immediately. Such artefacts were formed in the intracristae space and had completely different morphology and size than those present in the mitochondrial plasma of chondrocytes. They appeared as dots, filaments, microtubes, parallel arrays forming sheets, or coiled filaments arranged to helical structures; but they remained within the size of the intracristae space. They were found in neurons (Pena 1980); also similar structures have been described in various other kinds of tissues (Barastegui and Ruano-Gil 1984; Hall and Crane 1971).

In the present study on horse cartilage, such artefacts can largely be excluded, since samples were processed directly after harvest, and in addition articular cartilage is not as sensitive to changes in the environment as it is the case for neurons or muscle, since chondrocytes are embedded within a dense protecting matrix (Nürnberger et al. 2006b) and are marginally physiologically active. Further, samples of human articular cartilage analysed in this and previous studies (Nürnberger et al. 2006a, b) taken from femoral head replacement surgery did not show any crystals even though they were processed at different time points after surgery (immediately up to several days). Fixation artefacts should also be excluded, since the crystals were present in samples of the same individuals after treatment protocols by means of two different methods (histology and electron microscopy) and crystals were not found in cartilage or other samples of non-equine cartilage processed in the same way. In addition, the size of the chondrocyte crystals (several µm) stretching almost through the whole cell indicates that not only the crystals but also the mitochondria had to grow to a multiple of their original size. It is unlikely that chondrocytes which have a low metabolic activity are capable to synthesize and accumulate proteins of this amount in the course of sample preparation until fixation is completed.

The number of crystals per section varied from none up to six that were distributed singularly or in groups in one or few mitochondria, while most mitochondria of the cells were normal-sized and did not contain any crystals. This raises the question why protein aggregates are not regularly distributed over all mitochondria of one cell, but they accumulate in specific ones. It is a known fact that alterations concern only one or few mitochondria per cell, such as the enlargement to giant mitochondria (Tandler and Hoppel 1986). This individual reaction of mitochondria is probably related to the fact that cells may contain different subpopulations of those respiratory organelles, which vary in morphology (small globular or elongated), production of reactive oxygen species, or calcium content and membrane potentials (Banerjee et al. 2015; Kuznetsov et al. 2006). Membrane potentials are related with the trans-membrane transport mechanisms, such as inner and outer membranes transport TIM and TOM (Pfanner and Meijer 1997). A malfunction of one of the transport channels could be the reason for such accumulation: either by excess import of proteins or when proteins produced in the mitochondria exceed the discharging capacity towards the cytoplasm, similar to the experimentally induced IgG-accumulation in the endoplasmic reticulum of the Chinese hamster ovary cells after disequilibrium of IgG protein synthesis and transport to the Golgi apparatus (Hasegawa et al. 2011). Also the voltage-dependent anion channel (VDAC) is located in the outer mitochondrial membrane and responsible for transport of small molecule and ions (Lemasters and Holmuhamedov 2006). VDAC could also be responsible for protein accumulation inside the chondrocytes mitochondria, which is supported by the fact that it is voltage-dependent and mitochondrial subpopulations differ in their membranes potential (as discussed above). Inhibition of VDAC results in general mitochondrial suppression and is induced by circumstances which were also reported in relation with crystal formation in mitochondria such as the influence of ethanol (Voelz 1968), anoxia and hypoxia (Shiraki and Neustein 1971). Hypoxia is also present in cartilage and may exceed a certain limit in specific cartilage regions and during growth, leading to closure of the VDAC and accumulation of small proteins starting to crystallize in the mitochondria due to stochastic reasons. Considering the size of the crystals and the hypothesized loss of functionality of the mitochondria, the question arises whether the cells and the cartilage are still intact. The lack of interference with the vitality of the chondrocytes is probably due to an increased glycolysis, which is also part of the normal physiological condition in the unvascularized cartilage tissue (Nürnberger et al. 2006b).

In order to obtain further information about the nature of the crystals, fourteen histochemical staining methods were performed. Most intensive staining was achieved with anionic dyes such as Movat and Masson Goldner trichrom, further AZAN, GRAM, Giemsa and toluidine blue. Little stain affinity showed MSB and HE but not at all with Alcian blue, Safranin O, PAS, BBS and Picro Sirius red. This staining reactivity is similar but not identical to the well-characterized Reinke’s crystals of Leydig cells. Reinke’s crystals also have affinity to the anionic acid fuchsin in trichrom and GRAM staining (Mesa et al. 2015). PAS, in contrast, showed to some degree staining of Reinke’s crystals but did not stain crystals in the mitochondria, indicating the absence of polysaccharides. This shows that crystals differ in their composition and that equine chondrocytes crystals are not glycosylated proteins or they are only to a small extent.

The analysis of elements performed in this study revealed the presence of nitrogen, which is a typical component of proteins. However, it was not possible to achieve any further identification of a specific substance. The lack of sulphur and phosphor excludes nucleic acid. Sulphur was, however, found around the crystal and in the ribosomes at the ER and in the cytoplasm. This signal could probably derive from cystine of the ribonucleic acid of ribosomes. Similarly, Fourier transform analysis of the periodic function of the lattice has proven a regular arrangement of the substructure that is typical for protein crystals. The parameters of the unit cell could, however, not be identified completely.

Due to the specific activity of some cell types, especially hormone-expressing cells, it has been possible to identify in such cases the composition of the crystals by immunostaining. Therefore, it is known that Reinke’s crystals in the Leydig cells contain the androgen-specific steroidogenic enzymes 3β-hydroxysteroid dehydrogenase (3β-HSD (Mesa et al. 2015) and β-cells in developing pancreas insulin crystals (Riopel et al. 2014). It is further known that under the systemic disorder histocytosis macrophages and dendritic cells accumulate immunoglobulins (Dogan et al. 2012). Articular chondrocytes do not have a particularly increased enzymatic or synthetic activity, but are challenged with a hypoxic and load bearing environment. It is therefore possible that either hypoxia or mechanical stress are related with crystal formation, leading to a hyperactivity or disequilibrium of proteins with changing load conditions during growth or higher activity in young individuals. Hormonal reasons seem less likely to be influential, since crystals did also appear in some adult horses to a certain extent. This supports the hypothesis of an imbalanced production and mitochondrial transport—as discussed before. The fact that horses are heavy and athletic animals with especially high challenge in both static and dynamic mechanical stress on their joints may explain why crystals have not been found in other species so far. The higher frequency of crystals in younger individuals suggests a correlation between mechanical stress and the location and frequency of crystals. Thinner cartilage areas with elongated chondrocytes in the superficial zone bear more crystals than thick zones with rather spherical chondrocytes up to the surface. Both shape and orientation of chondrocytes are closely related to collagen alignment, and load deformation of the collagen network is transferred to the cells. In a study in foals, cartilages of two sites of the metacarpophalangeal joints were compared in terms of their changes in collagen architecture upon mechanical loading (Brama et al. 2009). One of the sites was isolated from an area challenged dynamically during jumping and galloping, and the other one from an area mainly loaded during standing. Dynamic, peak-loaded sites revealed higher changes than the constantly loaded one. The peak-load sites correspond morphologically to the equine cartilage areas with high crystal occurrence, which supports the theory that crystal formation is related to dynamic mechanical conditions. Further support is given by the fact that highest changes in collagen architecture (parallelism) during loading were detected in the upper cartilage area (Brama et al. 2009), which was also the area of highest crystal incidence in the current study. The mechanical stress that induces crystal formation could not only activate mitochondrial pathways but it also influences both the transport mechanism and the orientation of the crystals. The latter is supported by experimental results on shear-induced self-assembly of proteins into fibrils studied by atomic force microscopy and on high-stress shear-induced crystallization of polymers monitored by in situ X-ray scattering (Greving et al. 2012). Further indications for a biomechanical induction come from the conducted chondrocyte transplanting study. Areas adjacent to the defects bore crystals with especially large diameters. Furthermore, defects treated with in vitro stimulated constructs (Ponomarev et al. 2014) contained chondrocytes with crystals, but not those which were treated with statically cultured chondrocytes on a biomaterial (Nürnberger et al. 2013b).

## Conclusion

In this study, protein crystals inside mitochondria of equine chondrocytes are described in terms of their structural grid parameter and their histochemical characteristics. These crystals have presented special characteristics in terms of their size and appearance in chondrocytes, which are cells with low metabolic activity under no particular hormonal influence. The samples in this study presented no previous history of articular cartilage disease. Some of the horses were, however, part of a study on cartilage regeneration. The distribution of the crystals suggested a relation to areas of high mechanical stress, which could locally be the case in defects and in transplanted areas. Crystals were found especially in yearlings, suggesting also a developmental component, probably due to the anatomical changes and activities during growth. Blockade of mitochondrial transport mechanisms is a possible underlying mechanism for protein accumulation. Further studies on the composition of the crystals are necessary to understand the origin and reason for their development and physiological correlations.

## Electronic supplementary material

Below is the link to the electronic supplementary material.

Fig. S1 TEM images of crystals showing different characteristics.** a** Crystal stretching almost through the whole cell appearing homogenously dense in this magnification and orientation.** b** Three crystals with hexagonal profile of four long and two short sides and an inner striated structure. Medium dense matrix and a double membrane surround them, either alone, or in pair. c Rhomboidal cross section through a crystal surrounded by a double membrane and several cristae-like membranes arranged in a network-like form (*arrows*).* Scale bars* **a** 2 µm,** b** 500 nm and** c** 1 µm (TIFF 1340 kb)

Fig. S2 Histochemical stainings** a** MSB** b** HE that did hardly (*arrows*), or** c** PAS,** d** Alcian blue,** e** Safranin O,** f** BBS pH10 not stain the crystals in paraffin sections.* Scale bars* are 50 µm (TIFF 9112 kb)

Fig. S3 Masson Trichrom stainings of a cartilage region in transition to the periost showing that crystals preferentially appear in the superficial cartilage region but not in the overlapping periost.* a* Overview of the whole cartilage depth. The brackets indicate the area of magnification of** b**–**d**.** b** Detail images of the periost with elongated cells in between oriented collagen tissue.** c** Upper region of the hyaline tissue with many large crystals inside the cells; some of them are indicated with *arrows*.** d** Deep cartilage region with large cells but hardly any crystals inside.* Scale bar* **a** 50 µm,** d** (representative also for** b** and** c**) 20 µm (TIFF 15526 kb)
