# Supplementary figures and images for: Giant crystals inside mitochondria of equine chondrocytes
Source: Histochem Cell Biol. 2016 Dec 24;147(5):635–49. doi: 10.1007/s00418-016-1516-6 (PMC5400799; doi:10.1007/s00418-016-1516-6)

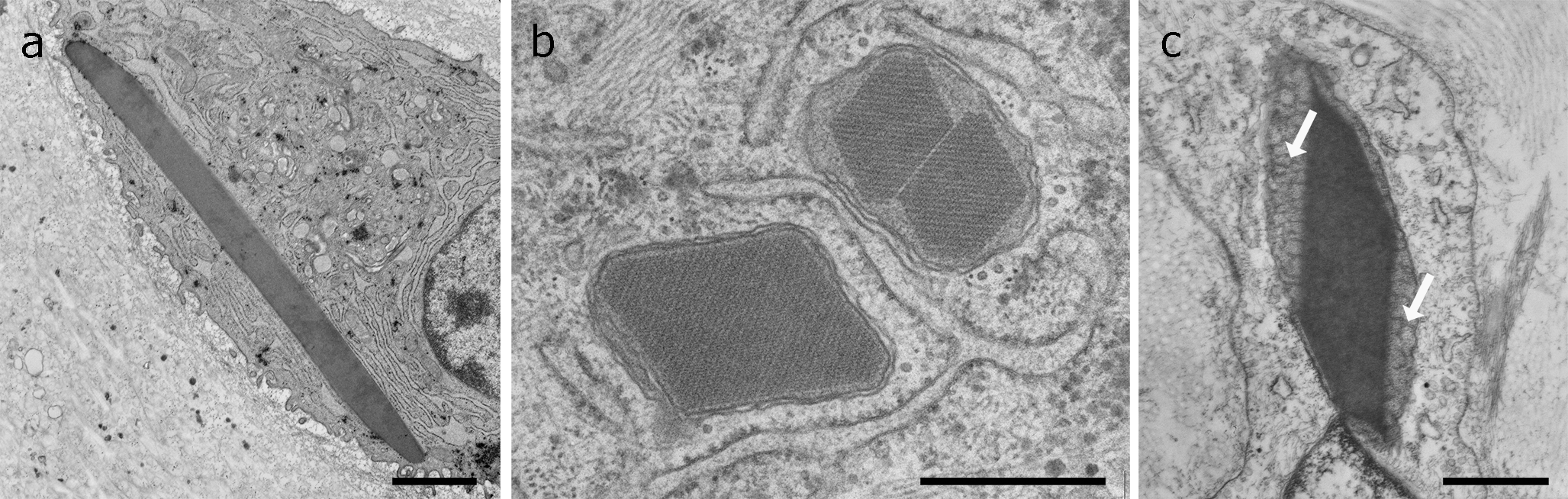

Supplement: Supplementary file 1 — TEM images of crystals showing different characteristics. a Crystal stretching almost through the whole cell appearing homogenously dense in this magnification and orientation. b Three crystals with hexagonal profile of four long and two short sides and an inner striated structure. Medium dense matrix and a double membrane surround them, either alone, or in pair. c Rhomboidal cross section through a crystal surrounded by a double membrane and several cristae-like membranes arranged in a network-like form (arrows). Scale bars a 2 µm, b 500 nm and c 1 µm (TIFF 1340 kb) [file 418_2016_1516_MOESM1_ESM.tif]

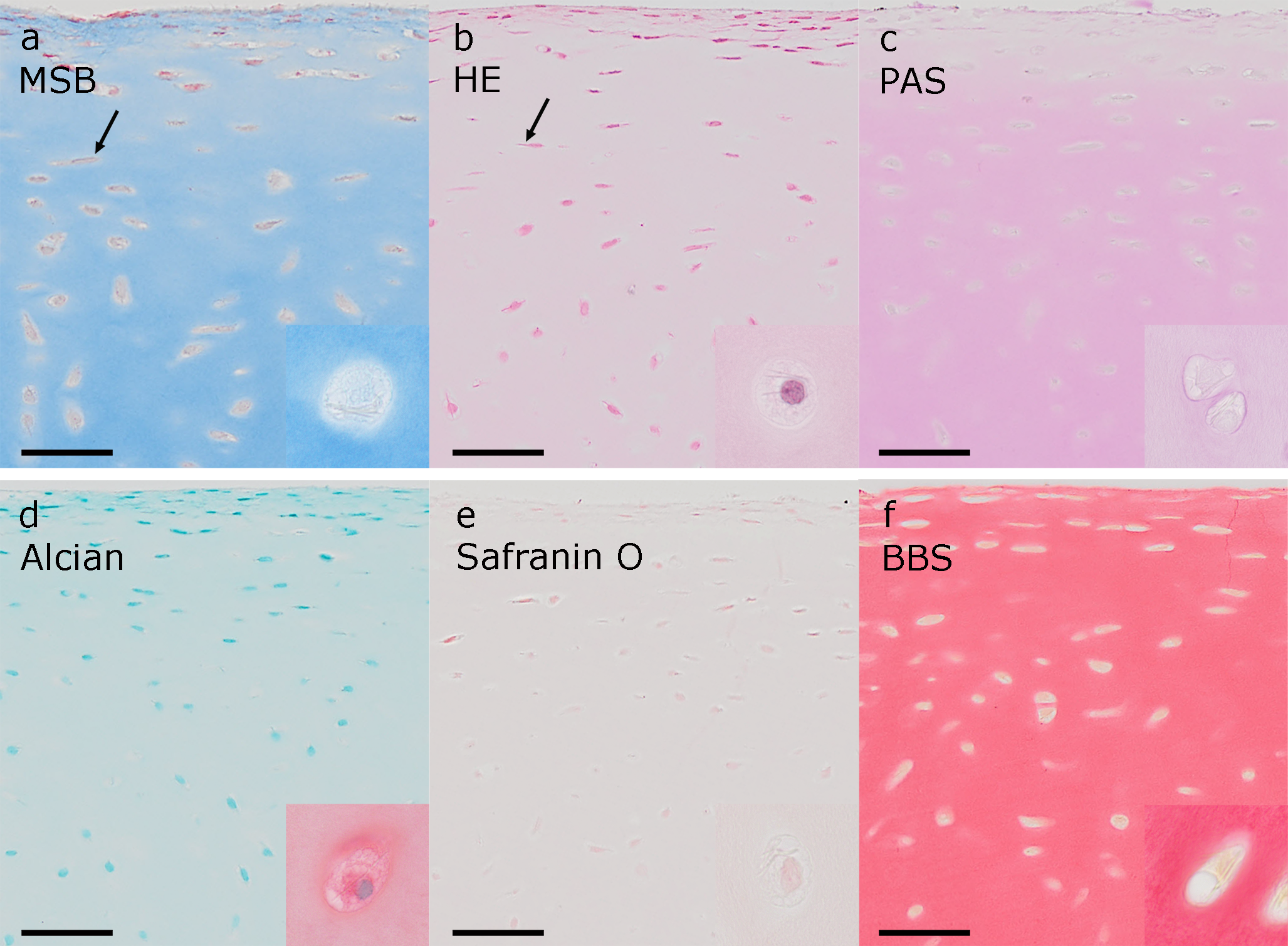

Supplement: Supplementary file 2 — Histochemical stainings a MSB b HE that did hardly (arrows), or c PAS, d Alcian blue, e Safranin O, f BBS pH10 not stain the crystals in paraffin sections. Scale bars are 50 µm (TIFF 9112 kb) [file 418_2016_1516_MOESM2_ESM.tif]

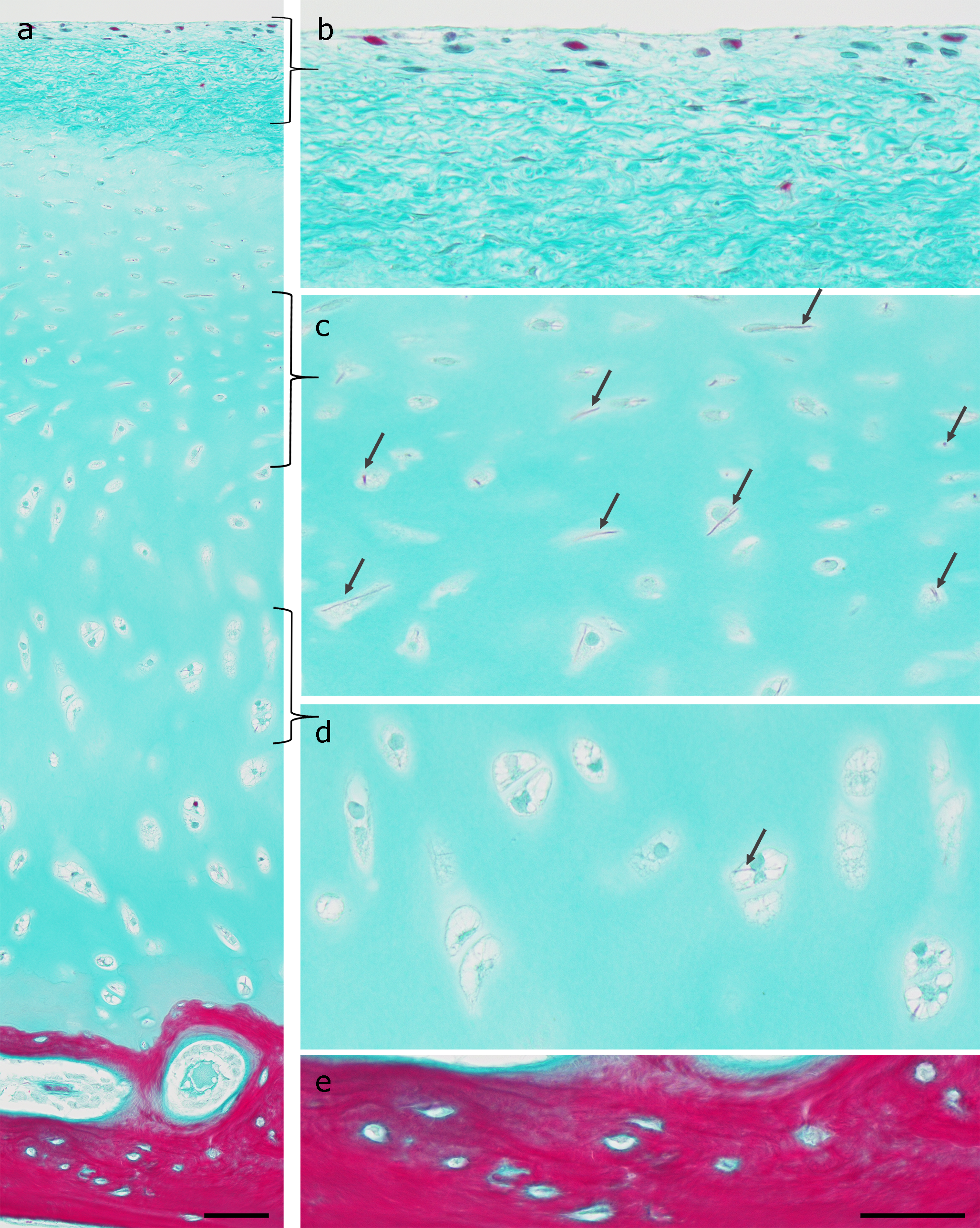

Supplement: Supplementary file 3 — Masson Trichrom stainings of a cartilage region in transition to the periost showing that crystals preferentially appear in the superficial cartilage region but not in the overlapping periost. a Overview of the whole cartilage depth. The brackets indicate the area of magnification of b–d. b Detail images of the periost with elongated cells in between oriented collagen tissue. c Upper region of the hyaline tissue with many large crystals inside the cells; some of them are indicated with arrows. d Deep cartilage region with large cells but hardly any crystals inside. Scale bar a 50 µm, d (representative also for b and c) 20 µm (TIFF 15526 kb) [file 418_2016_1516_MOESM3_ESM.tif]
